# First tracheal ring fracture in a complex thyroid surgery

**DOI:** 10.1016/j.ijscr.2019.12.012

**Published:** 2019-12-13

**Authors:** Raja Husain, Asayil Alnasser, Mohammed Al Duhileb, Tariq Madkhali

**Affiliations:** a King Fahad Hospital of the University, College of Medicine, Imam Abdulrahman Bin Faisal University, Dammam, Saudi Arabia; b Department of Surgery, King Fahad Specialist Hospital, Dammam, Saudi Arabia

**Keywords:** Thyroidectomy, Tracheal injury, Graves' disease

## Abstract

**Introduction:**

Tracheal injury is a rare complication of thyroidectomy that endocrine surgeons might face during or after the surgery. It accounts for less than 1 % of all thyroidectomy complications.

**Case presentation:**

A 48-year-old who presented with hyperthyroidism signs and symptoms, diagnosed with Graves’ disease. Patient underwent total thyroidectomy after failure of the medical management that ended in first tracheal ring laceration. Tracheal laceration discovered intraoperatively and repaired with primary closure.

**Discussion:**

Tracheal injury is one of the rare complications of thyroidectomy. It can be discovered intraoperative or postoperative. Tracheal injury can be managed conservatively or surgically depending on the size of the injury. But, the early diagnosis of it can lead to better outcome and decrease the mortality and morbidity.

**Conclusion:**

Tracheal injury can be prevented by increasing the awareness of its presentation and its risk factors.

## Highlights

• Graves’ disease treated by one of three approach which are pharmacological, ^131^I-radiotherapy, or thyroidectomy.
• Methimazole and Carbimazole are considered the first line of treatment for Graves’ disease.
• Most of thyroidectomy complications are rare and transient.
• Tracheal injury accounts for less than 1 % of all thyroidectomy complications.

## 1 Introduction

Thyroidectomy is one of the common and safe procedures done by the endocrine surgeons to treat both benign and malignant thyroid diseases, with very low complications rate. However, there are some rare complications that might suddenly appear during thyroidectomy, tracheal injury is one of them. Tracheal injury accounts for less than 1 % of all thyroidectomy complications. It is usually recognized and repaired intraoperatively, however late recognition can lead to serious and life-threatening complications such as tracheal necrosis and contamination [1,2]. This work has been reported in line with the SCARE criteria [3].

## 2 Case report

A 48‐year‐old gentleman, was medically free, presented to the endocrine clinic with history of palpitation, heat intolerance, fine tremor and unintentional weight loss. On September 2018, the patient was diagnosed with Graves’ disease and was started on Propylthiouracil (PTU) and Propranolol since that time. The patient was not responding to medical treatment and was referred to our endocrine surgery clinic for total thyroidectomy.

**Fig. 1:**
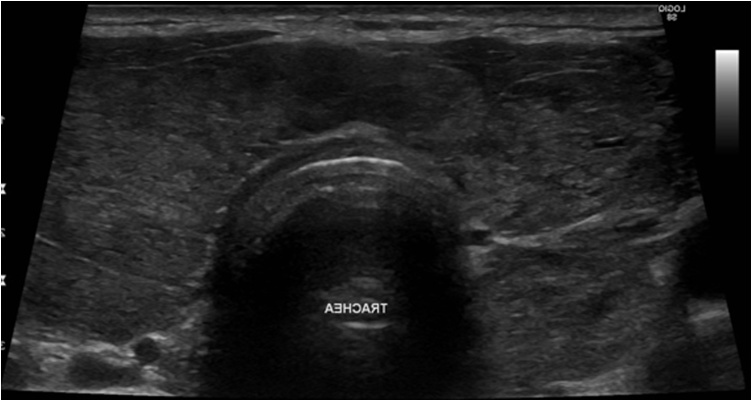
Ultrasound of the neck.

On physical examination, there were exophthalmos and chemosis (Graves’ ophthalmopathy). The neck examination showed diffuse swelling at the thyroid anatomical location, more on the left side, firm, not tender with no retrosternal extension. There was no cervical lymphadenopathy, no neck rash or scars. (Fig. 1)

Pre-operative laboratory investigations, FT3 = 11.27 (2.63–5.7) pmol/L, FT4 = 13.44 [10–20] pmol/L, Anti-thyroglobulin Antibodies was negative, and Anti-thyroid peroxidase Antibodies was positive.

During the total thyroidectomy, the surgery was complicated by first tracheal ring fracture. A small tracheal laceration that happened on the right side between the cricoid cartilage and first tracheal ring during shaving the thyroid from the trachea. Interestingly, the tracheal injury was repaired with 3 Prolene stiches.

## 3 Discussion

Graves’ disease is an autoimmune disease that affect thyroid gland mainly. It results from overproduction of thyroid hormone which caused by stimulation of the thyroid gland through binding of pathogenic antibodies thyroid receptor antibodies (TRAb) which also called thyroid stimulating immunoglobulin (TSI) that synthesized by the B lymphocyte to the thyroid stimulating hormone (TSH) receptors. It is characterized by hyperthyroidism, goiter due to the effect of TSI on the thyroid gland, and in some patients, graves’ ophthalmopathy that might include exophthalmos, chemosis, lid-lag and lid-retraction as a result of the inflammation, and proliferation of the extraocular muscles and retro-orbital connective and adipose tissues secondary to the effect of TSI and cytotoxic T lymphocyte. It is the most common cause of hyperthyroidism in the iodine sufficient areas, account for 60–80 % of all cases. Many studies found that the incidence is around 24.8 patients per 100,000 per year. Graves’ disease is more common in female, suggested by some data that reports approximately 2 % of female and 0.2 % of male develops graves’ disease during their life. [[4], [5], [6], [7], [8]]

### 3.1 Management

Hyperthyroid due to Graves’ disease treated by one of three approach which are pharmacological, ^131^I-radiotherapy, or thyroidectomy. The choice of appropriate approach depends on many factors, but the main goals of treatment are to restore thyroid function, prevent recurrence and hypothyroidism. The first approach is antithyroid drugs: propylthiouracil (PTU), and thionamide derived antithyroid drugs include methimazole and carbimazole are considered the first line of treatment for Graves’ disease. These drugs decrease level of thyroid hormones (T_3_ and T_4_) by inhibiting Thyroid peroxidase (TPO) action which play a role in the synthesis of thyroid hormones. In addition to that, PTU prevent the peripheral conversion of T_4_ to T_3._ American Thyroid Association (ATA) and American Association of Clinical Endocrinologists (AACE) guidelines suggested that all the patient who are candidate for antithyroid drugs should be treated with methimazole except women in the first trimester of pregnancy, during thyroid storm, and patients who develop adverse events secondary to methimazole. The main adverse events of antithyroid drugs are hepatitis, jaundice, urticaria, rash, lupus like syndrome, and agranulocytosis [[9], [10], [11]].

The second approach to treat patients with Graves’ disease is ^131^I-radiotherapy which acts by destruction of thyroid tissue leading to hypothyroidism in most of the patients. It is indicated in the patients who are not candidate for antithyroid drugs, or who want definitive treatment without the risk of surgery. However, it is contraindicated in pregnancy and breast feeding [9,10].

The third modality to treat the patients with Graves’ disease is thyroidectomy which is the definitive treatment for these patients. But it is the least chosen modality due to the risk of complications although the rate of recurrence of Graves’ after surgery is 0 %. The complication rate after surgery is more drastic than the other modality, however, the complications are rare and transient in most of the cases. The complications might include hypocalcemia, recurrent laryngeal nerve injury, hypoparathyroidism, hematoma, and wound infection. It is indicated when there is failure in medical treatment, refusal or low uptake of ^131^I, large goiter that cause compressive symptoms, malignancy suspicion, Graves’ ophthalmology, and pregnancy planning in the near future. Regarding the extant of thyroidectomy, total thyroidectomy is better than sub-total, although many recent studies reported that there is no difference in the complications rate expect the rate of recurrence is higher in sub-total thyroidectomy [5,7,9,10,12].

Preparations should be considered before surgery to decrease the risk of developing some complications that include euthyroid state before surgery to decrease risk for thyroid storm. This could be achieved by using antithyroid medication 1–3 months before surgery, however, there are some patient required urgent surgery, in these cases, β-blocking drugs, potassium iodine, dexamethasone, and cholestyramine are recommended. Administration of potassium iodine for 10–14 days also can be used to decrease thyroid vascularity that minimize intraoperative blood loss [13,14].

One of the rare complications of thyroidectomy is tracheal injury. It accounts for less than 1 % of all thyroid complications. There are many preoperative, intraoperative, and postoperative risk factors that can lead to tracheal injury that includes goiter. Many studies reported that continuous compression on the trachea by large goiter can lead to tracheal weakness and tracheomalacia which is define as weakness of the tracheal cartilages and hypotonia of the elastic elements. Female gender is considered another risk factor for tracheal injury. Regarding intraoperative causes include prolong intubation, high cuff pressure that lead to vasoconstriction, decrease blood supply to trachea, and subsequently tracheal damage, inability to recognize thyroid plane of dissection in the case of multi-nodular goiter, and lastly uncontrolled postoperative cough [2,15,16].

**Fig. 2:**
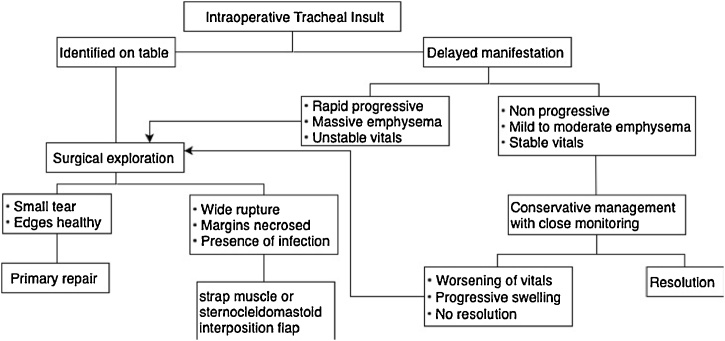
management of tracheal injury [18].

Diagnosis of the tracheal injury can be intraoperative or postoperative, the common signs of tracheal injury are Pneumomediastinum and subcutaneous emphysema. Tracheal injury can managed conservatively or surgically depend on the size of the injury, in some cases delay discovery of the injury can lead to tracheal necrosis which should be managed by debridement to revitalize the trachea. Small injury without air leakage in the mediastinum managed conservatively by bed rest, antibiotics, and cough suppression followed by primary closure of the wound by suture if the conservative management failed. In contrast large wound or contaminated wound managed by different methods but the most effective method is closure with suture with or without muscle flap, (Fig. 2) [[17], [18], [19]].

## 4 Conclusion

Tracheal injury is a rare but life-threatening complication of thyroidectomy, that can be managed easily if discovered early. Indications of thyroidectomy should be studied very well before making any decision, although putting those rare complications in our consideration is another matter. Awareness about these rare complications, and their risk factors can help in preventing them and decreasing their mortality and morbidity.

## Declaration of Competing Interest

No conflict of interest to declare

## Sources of funding

no source of funding

## Ethical approval

Institutional Review Board approval. Case Report is presented anonymously.

## Consent

Informed written consent was taken from patient

## Author contribution

Raja Husain: writing the paper, submission

Asayil Alnasser: writing the paper

Mohammed Al Duhileb: writing and revision of the paper

Tariq Madkhali: revision of the paper

## Registration of research studies

Not Applicable (Case Report; not an interventional study).

## Guarantor

Mohammed Al Duhileb

Department of Surgery, king Fahad specialist hospital, Dammam, Saudi Arabia.

## Provenance and peer review

Not commissioned, externally peer-reviewed

## References

[1] Damrose E.J., Damrose J.F. (2009). Delayed tracheal rupture following thyroidectomy. Auris Nasus Larynx.

[2] Tartaglia N., Iadarola R., Di Lascia A., Cianci P., Fersini A., Ambrosi A. (2018). What is the treatment of tracheal lesions associated with traditional thyroidectomy? Case report and systematic review. World J. Emerg. Surg..

[3] Agha R.A., Borrelli M.R., Farwana R., Koshy K., Fowler A., Orgill D.P., For the SCARE Group (2018). The SCARE 2018 statement: updating consensus surgical CAse REport (SCARE) guidelines. Int. J. Surg..

[4] Pokhrel B., Bhusal K. (2019). Graves disease. [Updated 2019 Jun 3]. StatPearls.

[5] Mohan V., Lind R. (2016). A review of treatment options for Graves’ disease: why total thyroidectomy is a viable option in selected patients. J. Community Hosp. Intern. Med. Perspect..

[6] Cheetham T., Boal R. (2019). Graves’ disease. Paediatr. Child Health.

[7] Stathopoulos P., Gangidi S., Kotrotsos G., Cunliffe D. (2015). Graves’ disease: a review of surgical indications, management, and complications in a cohort of 59 patients. Int. J. Oral Maxillofac. Surg..

[8] Hussain Y.S., Hookham J.C., Allahabadia A., Balasubramanian S.P. (2017). Epidemiology, management and outcomes of Graves’ disease-real life data. Endocrine..

[9] Muldoon B.T., Mai V.Q., Burch H.B. (2014). Management of Graves’ disease: an overview and comparison of clinical practice guidelines with actual practice trends. Endocrinol. Metab. Clin. North Am..

[10] Bartalena L. (2013). Diagnosis and management of Graves disease: a global overview. Nat. Rev. Endocrinol..

[11] Burch H.B., Cooper D.S. (2015). Management of graves disease: a review. JAMA.

[12] Cipolla C., Graceffa G., Calamia S., Fiorentino E., Pantuso G., Vieni S. (2019). The value of total thyroidectomy as the definitive treatment for Graves’ disease: a single centre experience of 594 cases. J. Clin. Transl. Endocrinol..

[13] Piantanida E. (2017). Preoperative management in patients with Graves’ disease. Gland Surg..

[14] Nair G.C., C Babu M.J., Menon R., Jacob P. (2018). Preoperative preparation of hyperthyroidism for thyroidectomy - role of supersaturated iodine and Lithium carbonate. Indian J. Endocrinol. Metab..

[15] Al-Hijaj M., Al-Mansori S. (2012). Tracheal injury during thyroid surgery. Basrah J. of Surg..

[16] Tripathi D., Kumari I. (2008). Tracheomalacia: a rare complication after thyroidectomy. Indian J. Anaesth..

[17] Karakozis S. (2015). Management of complications after thyroid surgery. Hell. J. Surg..

[18] Devaraja K., Kumar R., Sagar P., Kumar R. (2018). Delayed presentation of tracheal injury after thyroidectomy—a case report. Indian J. Surg..

[19] Majeski James, Lynch W. (2013). Tracheal injuries diagnosed during thyroid surgery. Am. Surg..

